# A focus on cross-purpose tools, automated recognition of study design in multiple disciplines, and evaluation of automation tools: a summary of significant discussions at the fourth meeting of the International Collaboration for Automation of Systematic Reviews (ICASR)

**DOI:** 10.1186/s13643-020-01351-4

**Published:** 2020-05-04

**Authors:** Annette M. O’Connor, Paul Glasziou, Michele Taylor, James Thomas, René Spijker, Mary S. Wolfe

**Affiliations:** 1 College of Veterinary Medicine, Iowa State University, 1800 Christensen Drive, Ames, IA 50011-1134 USA; 2 Present Address: College of Veterinary Medicine, Michigan State University, East Lansing, MI 48824 USA; 3 Bond University, Robina, Queensland 4226 Australia; 4 US Environmental Protection Agency, Research Triangle Park, NC 27709 USA; 5 EPPI-Centre, University College London, London, WC1E 6BT UK; 6 Cochrane Netherlands, Julius Centre for Health Sciences and Primary Care, University Medical Centre Utrecht, University Utrecht, Utrecht, the Netherlands; 7 Medical Library, Amsterdam Public Health, Amsterdam UMC, University of Amsterdam, Amsterdam, the Netherlands; 8 US National Institute of Environmental Health Sciences, Research Triangle Park, Raleigh, NC 27709 USA

**Keywords:** Automation tools, Data abstraction, Data extraction, Evidence synthesis, Priority ranking

## Abstract

The fourth meeting of the International Collaboration for Automation of Systematic Reviews (ICASR) was held 5–6 November 2019 in The Hague, the Netherlands. ICASR is an interdisciplinary group whose goal is to maximize the use of technology for conducting rapid, accurate, and efficient systematic reviews of scientific evidence. The group seeks to facilitate the development and acceptance of automated techniques for systematic reviews. In 2018, the major themes discussed were the transferability of automation tools (i.e., tools developed for other purposes that might be used by systematic reviewers), the automated recognition of study design in multiple disciplines and applications, and approaches for the evaluation of automation tools.

## Background

This report summarizes the fourth meeting of the International Collaboration for Automation of Systematic Reviews (ICASR), which is an interdisciplinary group with a shared interest in maximizing the use of technology to aid the transfer of scientific research findings to practice and to inform decision-making. ICASR aims to develop the capability for conducting rapid, accurate, and efficient systematic reviews of scientific evidence. Each year, the organizing committee designs the meeting agenda to facilitate open exchange among participants, including (i) sharing of information on areas of investigation related to the automation of systematic reviews since the prior ICASR meeting, (ii) providing updates on previous ICASR meeting topics, and (iii) introducing new “theme/automation task” via talks by invited speakers. Previous ICASR meetings were held in September 2015 [1], October 2016 [2], and October 2017 [3]. A website provides other information about ICASR (https://icasr.github.io/about.html).

## The fourth ICASR meeting: scope

The overall goals of the fourth ICASR meeting were to:

1. Identify tools developed for other applications that might be repurposed for systematic reviews OR vice versa (a new theme in 2018).
2. Carry out automation of study design recognition, with an initial focus on randomized controlled trials (RCTs) (a new theme in 2018).
3. Conduct evaluations of automated tools (a theme discussed previously in 2017).
4. Determine how systematic review teams are currently using available automated tools (a theme previously discussed in 2017).

The theme and agenda were determined by the organizing committee which is involved in methods and production aspects of the systematic review community. The committee is diverse in discipline and focus (human health, social sciences, food safety, animal health, technology) which ensures a wide range of opinions are considered. Discussions about the agenda occur at monthly meetings. Whereas goals 3 and 4 had been examined in previous years, goals 1 and 2 were added for consideration at this meeting. During the meeting preparation, members of the organizing committee discussed having encountered tools that, although not designed for systematic reviews, could be used in this context; goal 1 was added to the agenda to explore the repurposing of other tools that might help to advance the efficient use of automation in systematic reviews. For example, machine learning tools used for electronic medical records, or tools used for extraction of data from PDF in other disciplines. The rationale for this addition was to leverage advances in other areas for systematic reviews, to avoid duplication of effort and speed progress. Further, discussions with research funders revealed that automation tools for systematic reviews could potentially assist the processes they use for selecting and tracking the outcome of research projects. Goal 2 was added as a result of new study classification tools that are now becoming more widely available. This type of automation has not received as much attention as has, for example, active learning for citation screening. However, study type classifiers (e.g., case control, RCT, etc.) built from large volumes of research have the potential to reduce citation screening workload in a more predictable way. In light of the adoption of such classifiers, this significant new goal was added to the agenda. The rationale for this goal is that study design is an essential feature that informs many downstream aspects of systematic reviews and therefore automated approaches to recognition of different designs would assist the production of reviews. For example, the risk of bias assessment for laboratory-based experimental studies, randomized control trial, and observational studies are very different, therefore automation recognition would enable direction of papers to different risk of bias assessment tools.

## Meeting agenda

The organizing committee invited approximately 50 participants, including users of summarized research, methodologists, funders, technologists, developers/coders, and computer scientists. The participants were identified as either prior participants at ICASR meetings or suggestions from the organizing committee members. When the invitations were distributed, the invitees were requested to suggest alternatives if they or the organization they represented were unable to attend. The international group of invitees from public and private sectors was selected to ensure a diversity of perspectives (funding groups, researchers, research synthesizes, methodologists, etc.) and disciplines (clinical health, public health, environmental sciences, food production, climatology, social sciences, text analytics, machine learning and human-computer interaction, etc.) about research synthesis and to limit the number of participants from any one particular company or institution. Thirty-five participants attended the one-and-a-half-day meeting. The agenda was divided into sessions that included presentations by selected participants, large group discussions, and small group discussions on focused topics. At the end of the meeting, time was set aside to discuss future directions for the field and ICASR sustainability.

### Common themes that emerged during the meeting

The overarching concepts presented and discussed for each of the individual scientific sessions at the fourth ICASR meeting are summarized below. The three focused session topics were as follows:

- Approaches to evaluating tools for automation of systematic reviews (goals 3 and 4)
- The transferability of automation tools (goal 1)
- Automated recognition of study design (goal 2)

#### Approaches to evaluating tools for automation of systematic reviews

As in prior years, the importance of evaluation of automated tools was a recurring theme and major discussion topic (goal 3). The group considers this a critical issue, as without robust evaluations, potential users of new technologies will be hesitant about adopting them [4]. The discussion focused on how to evaluate tools, including whether the method of evaluation would be the same for all automation tasks or tailored to specific circumstances. Three possible approaches to tool evaluation were presented:

- Evaluation by human discrimination—comparing against a manually performed review.
- Evaluation through problem benchmarks—comparing against a standardized dataset.
- Evaluation by peer confirmation—comparing against results from different automation tools.

Deciding which automation tool to use might depend on the complexity of the systematic review task; for example, is it a low inference task (e.g., extracting the article’s title) or a high inference task (e.g., identifying the study design). Similarly, automation tools should be assessed based on their performance of low and high inference tasks. A low inference task might require only peer confirmation as an evaluation approach (i.e., just a simple comparison of which tool is most accurate). Alternatively, a high inference task, such as study design identification (e.g., observational study), would likely require an evaluation approach through problem benchmark, that is, a gold-standard dataset developed by multiple external experts. Importantly, the concepts of low inference and high inference automation tasks should not be interpreted as synonyms for “easy” versus “hard” automation tasks. For example, the extraction of a measure of association from a table, such as a risk ratio comparing disease risk in groups in an RCT, might be considered a low inference task because it is a single numerical target; however, this task is challenging technically due to the complexities of working with tables in documents. Further, low inference and high inference tasks are not necessarily universally constant. For example, extraction of a risk ratio from an observational study that reports four models with different variable selection and confounder adjustment approaches is likely a high inference task even though the target for automated extraction is a numerical measure of the association.

Another point raised is the need to incorporate an assessment of the user interface into the evaluation of automation tools. Standard metrics of automation tool evaluation, such as accuracy, recall, and precision, do not capture either the user experience or the ease/difficulty of integrating tools into the systematic review workflow. Discussions also pointed toward a more cost/benefit-focused model in which clear expectations are prospectively defined. Further, insight needs to be provided on what costs would be acceptable in a broad sense (cost can be actual time/personal costs but can also be the desired “confidence” in the end result). Developers need to more clearly articulate the human–machine level of cooperation and interaction expected for the product. Currently, review teams with little experience using automation tools might expect a fully automated experience, while the developers planned for a significant degree of human supervision or assistance.

Another topic discussed was the importance of appropriate external evaluation of tools by users rather than developers. External evaluation is extremely important because it provides an independent assessment of the tool. If independent external evaluations are consistent with the developer’s evaluation, this consistency increases confidence in the tool. However, the external evaluation of a tool for a purpose(s) for which the tool was not intended is a particular concern for developers. External evaluators should provide clear justification for the hypothesis that the tool could perform the novel task, particularly if the method of use differs from that recommended by tool developers. For example, given the vocabulary differences in authors’ descriptions of RCTs and observational studies, and in the factors that contribute to the accuracy of RCT classifiers (terms such as blinding, allocation, randomization), an a priori rationale could explain why an RCT classifier might not effectively classify associational observational designs such as case-control studies or cohort studies. The group discussed whether a classifier built on the vocabulary of RCTs in the medical field might not have the same applicability within other disciplines, such as sociology and food production, due to differences in how the fields might describe RCTs. External evaluations should be hypothesis-driven, carefully planned, and transparently communicated. Some developers indicated that they might be open to discussing the rationale for external evaluations during the development of the external evaluation protocol. However, this approach is not likely to be universally adopted because developers might not have the time to be involved in all potential external tool evaluations and there is an apprehension that such communication would compromise the independence of the external evaluation.

Additionally, systematic reviewers need to recognize and appreciate developers’ unease about the external evaluation of automation tools for unintended purposes, especially in other research domains. Developers also need to recognize the need to use reviewers from different research domains to document the limitations of available tools. The concern is that funders will extrapolate the reported accuracy of tools developed by Cochrane (a network of researchers and practitioners that produce systematic reviews according to a specific, published methodology) or similar groups for clinical trials as applicable to other review areas such as climate change, environmental health, food production, and biomedical research.

In summary, the critical discussion points for the session on the evaluation of automation tools were as follows:

- Different evaluation approaches might be more suitable for tasks that require different levels of human involvement (e.g., high inference tasks)—which currently require a high degree of human knowledge, such as the evaluation of observational study design—and would benefit from evaluations using internationally developed gold-standard datasets, whereas low inference tasks might be sufficiently assessed using peer confirmation.
- The extent of human–machine interaction anticipated by developers for the automation tool should be transparently communicated to the intended end-users. Metrics for assessing the human–machine interaction should be included in the assessment.
- The external evaluation of automation tools for unintended purposes should have a hypothesis-driven basis, be carefully planned, and transparently communicated so as not to mislead.

#### Transferability of automation tools

Another major theme of presentations and discussions at the fourth ICASR meeting was the potential for tools developed by systematic reviewers to serve purposes for other applications and vice versa (goal 1). Areas identified as having the most promise for transferability of technology-assisted systematic review tools were the scientific publication sector and the grant review process. For example, systematic review tools designed for the extraction of data elements could be used to check for comprehensive reporting when scientific articles are submitted for peer review. Another urgent need common to both scientific publication and systematic reviews is the capability to detect internal inconsistency of information within a manuscript, such as finding that the sample size or the measure of association is not consistent throughout the manuscript, or that slightly different numbers are reported in the abstracts, tables, or text. Although detection of the “correct” number may be beyond the scope of the tools, it is perhaps feasible to use automation to flag inconsistencies (or “anomalies”) that would require further human validation.

Funding agencies might be another potential group with interest in repurposing systematic review tools to use them to check submitted research protocols against comprehensive reporting standards, to detect for the duplication of projects compared with published or previously funded work, and to compare the protocol with the final report.

#### Automated recognition of study design

Speakers from diverse disciplines gave presentations on the automation of study design recognition and the use of tools in nontraditional research synthesis such as funding agencies (goal 2). With regard to RCTs conducted in human populations, Cochrane has a well-developed and accurate process for identifying randomized trials in publications as they become available through electronic databases^1^^,^^2^. Cochrane is now developing a classification system for clinical studies that applies tags for the population, intervention, comparison, and outcome (PICO) areas, which allows researchers to search for studies with specific PICO attributes. To standardize the search terminology, Cochrane has developed an ontology [5] using the vocabularies from various sources including SNOMED CT, MeDRA, RxNorm, and MeSH.^3^ Cochrane took this subset of vocabularies that are globally used and structured them within the PICO framework. As new groups begin to annotate their content, vocabulary gaps are exposed, which creates a constant need to update the ontology. At the time of this meeting, the tool was still in development and publicly accessible content is limited to the ontology definition^4^ and a term-and-PICO-search tool^5^.

Areas outside of clinical health research for which automated tool development would be useful include tools to recognize associational observational studies, experimental animal studies, studies that estimate a single group characteristic such as disease incidence, and studies of diagnostic tests. It was noted that groups working outside of clinical health have found machine-learning classification algorithms useful for screening large volumes of research captured by the necessarily broad searches for these areas. However, tools for data extraction or automated literature searches of databases other than publicly available databases, such as those maintained by the United States-based National Library of Medicine, do not appear to be available. Unfortunately, judgment-specific tools (e.g., RobotReviewer) do not seem to translate well to non-clinical health applications, although a lack of training data inhibits both tool production and comparative evaluation. Interestingly, much of this knowledge appeared anecdotal, perhaps partly because of reservations about publishing assessments of tools for tasks for which they were not designed, as discussed earlier in this summary.

### Goals of prior ICASR meetings and future of ICASR

Although many researchers have made progress on the development and adoption of automation tools for systematic review, the enormity of this task becomes more evident with each meeting. The meetings have been successful in bringing together a community of researchers and sharing knowledge of what tools are available and are being used (goal 4) throughout the entire meeting. The meetings have also been successful in identifying new topics of relevance to the automation of systematic reviews, and the diversity of disciplines involved (environmental, toxicology, food supply and production, clinical health, public health, and behavior sciences) has grown since the original meeting [1]. This expansion of disciplines has highlighted that many disciplines are facing the same issues related to evidence synthesis—the vast amount of data available, the desire to shorten the time for conducting the review, and the aim to reduce costs. A discussion of the future of ICASR noted that a collaboration between ICASR and the EU COST Action “EVBRES,” may occur in the next few years. One of the goals of EVBRES is to promote the use of systematic reviews before new research is conducted. To be able to do this in a timely manner, both rapid review methodology and automation are needed. The combined knowledge of ICASR therefore can be employed for EVBRES, which can be used to promote the implementation of the various tools within its broad network.

## Conclusion and future goals

The fourth ICASR meeting was successful in reviewing the status of existing systematic review automation tools and identifying new tools that are being built. The meeting also provided an excellent opportunity to bring together a diverse group of people working in this area and to enable participants to discuss lessons learned, identify challenges, and gain perspectives from others in the same or different fields or roles (programmers, developers, reviewers, funders, and users). Although it is impossible to know the extent of interdisciplinary communication and collaboration that would have occurred without the annual ICASR meetings, the diversity of disciplines now involved in ICASR and the growth in interdisciplinary projects suggests that ICASR is realizing its intended purpose: to foster collaboration toward automation of systematic reviews and leverage the efforts of multiple communities for the benefit of all. Outcomes of the third ICASR meeting included establishing an ICASR website, hosting successful hack-a-thons involving members of the ICASR community, developing several collaborative publications, sharing of knowledge, and initiating new research teams. With each year, collaborations established between ICASR participants between meetings become stronger and more frequent.

An outcome of the fourth ICASR meeting was a decision to use the Open Science Framework, Twitter, and Slack as the collaboration tool, dissemination tool, and communication tool, respectively. It was announced that the Western Norway University of Applied Sciences would host the fifth ICASR meeting in Norway combined with an evidence-synthesis hack-a-thon and that a “local” ICASR meeting would be held as part of the annual Cochrane Colloquium in Santiago, Chile, in October 2019.

## Data Availability

Not applicable.

## Abbreviations

API: Application programming interface
EPA: US Environmental Protection Agency
HAWC: Health Assessment Workspace Collaborative
ICASR: International Collaboration for Automation of Systematic Reviews
NIEHS: US National Institute of Environmental Health Sciences
PDF: Portable document format
PICO: Populations, interventions, comparisons, and outcomes
RCTs: Randomized controlled trials
SEED: Systematic EvidEnce Disseminator

## Footnotes

1 Noel-Storr A, Dooley G, Steele E, et al. (n.d.) An evaluation of Cochrane Crowd finds that crowdsourcing can help to address the challenge of information overload in evidence production (submitted). Journal of Clinical Epidemiology

2 Thomas J, Noel-Storr A, MacDonald S, Shemilt I, Elliott J, Mavergames C, Marshall I (n.d.) Machine learning reduces workload with minimal risk of missing studies: development and evaluation of the Cochrane RCT Classifier (submitted). Journal of Clinical Epidemiology

3 https://linkeddata.cochrane.org/linked-data-project/metadata-and-vocabularies

4 https://linkeddata.cochrane.org/linked-data-project/metadata-and-vocabularies

5 https://data.cochrane.org/pico-finder2/#/search
